# Walking in nature may improve affect but not cognition

**DOI:** 10.3389/fpsyg.2023.1258378

**Published:** 2024-01-05

**Authors:** Janet P. Trammell, Jennifer A. Harriger, Elizabeth J. Krumrei-Mancuso

**Affiliations:** Seaver College, Pepperdine University, Malibu, CA, United States

**Keywords:** nature, environment, affect, attention, cognition, memory

## Abstract

Beneficial effects of natural environments on affect have been consistently reported, but effects on cognition have been less consistent. We examined affect and cognitive performance in the domains of attention, working memory, executive function, and recall and recognition memory in a sample of 188 undergraduate participants who completed a walk in one of three environments: an outdoor nature environment, an outdoor urban environment, or an indoor (treadmill) environment. Supporting the hypotheses, the outdoor nature environment resulted in the greatest increase in positive affect and decrease in negative affect from pre-to post-walk. However, there were no effects of location on any cognitive measure. These results suggest that cognitive effects do not always occur in tandem with affective benefits. Possible explanations, including prior frequent exposure to nature in our participants and extremity of the natural environment, are discussed.

## Introduction

John Muir, one of the most influential nature conservationists in the 1800s, famously wrote that “In every walk with nature one receives far more than he seeks” (Muir, 1877). While Muir often wrote of the peace and inspiration that nature brings, it was not until the late 20th and early 21st century that scientific research into the benefits of nature for mental well-being began in earnest. Nature is a term that encompasses a wide variety of stimuli ranging from lush mountain rivers teeming with plant and animal life to dry, nearly lifeless and monotone landscape of deserts. The underlying commonality is a lack of human-made stimuli (with their accompanying noises and smells), such as buildings and machines. Research in this area, however, has primarily defined natural environments as those encompassing green and/or blue spaces (e.g., forests, parks, rivers) that contain plenteous vegetation and water and few human elements.

There is a wealth of literature regarding the beneficial effects of exposure to nature on well-being, such as increased positive and decreased negative affect across the lifespan (Bowler et al., 2010; McMahan and Estes, 2015; Norwood et al., 2019; Zhang et al., 2020). Such increases in positive affect (Berman et al., 2012) and decreases in negative affect (Watkins-Martin et al., 2022) even extend to those diagnosed with clinical depression. Further, contact with natural environments does not have to be long, with durations as little as 5 min resulting in improvements in positive and negative affect (Neill et al., 2019); however, “active lingering” in the environment can provide greater benefits (Li et al., 2019). While all types of nature exposure (e.g., viewing pictures or videos vs. actual physical immersion in a natural environment) appear to be beneficial, physical immersion has been shown to result in stronger effects than virtual nature (Kjellgren and Buhrkall, 2010; Browning et al., 2020). Further, the greater the tree cover in the environment, the greater the benefits (Jiang et al., 2015).

Likewise, there is general consensus that nature exposure is beneficial for cognition, particularly in measures of attention and working memory (see Ohly et al., 2016; Stevenson et al., 2018 for meta-analyses), although inconsistencies exist. Active engagement with the environment, such as directing attention toward trees (Lin et al., 2014) or observing and focusing on relaxing in the environment (Pasanen et al., 2018), has been shown to strengthen these cognitive effects; in contrast, both mindful and direct engagement strategies were not found to be effective (Macaulay et al., 2022). Additionally, cognitive and attentional benefits of nature exposure are not universally consistent; research has shown that they may be limited to tasks that involve moderate rather than high attention (Trammell and Aguilar, 2021) and may be partially explained by pre-test differences (Bowler et al., 2010). Nature-related improvements in cognitive performance in other domains, such as executive function (Trammell and Aguilar, 2021) and creativity (Palanica et al., 2019), have shown benefits in some tasks or situations but not others. Other measures of cognition, such as recall and recognition memory (Trammell and Aguilar, 2021), vigilance, impulse control, and processing speed (Stevenson et al., 2018) have not shown improvement as a result of nature exposure. To further elucidate past inconstant findings across cognitive tasks, we will examine the effects of environment on both moderate and high attentional tasks, executive function, and memory.

Theories regarding the mechanisms behind the affective and cognitive benefits of nature have focused on the restorative quality of nature. For instance, Attention Restoration Theory (ART) asserts that the involuntary yet low-key attentional demands, or “soft fascination” of these environments allows for depleted mental resources to recover by providing a respite from the more controlled and concentrated demands of typical urban environments (Kaplan, 1995; Berman et al., 2008), while the Stress Reduction Theory (SRT; Ulrich et al., 1991) proposes that nature exposure results in physiological improvements in stress, which relates to overall affective benefits and leads to improvements in cognitive tasks.

The purpose of this research was (1) to add to the literature regarding inconsistent effects of natural environments on various cognitive tasks and (2) to provide additional controls through the inclusion of two distinct control groups. One possible explanation for the inconsistent effects on cognition is the impact of being outside. Palanica et al. (2019) showed that in outdoor environments, creativity was similarly stimulated in both an urban and nature environment but was stronger for those viewing nature vs. urban stimuli in indoor environments, suggesting that simply being outside can be beneficial. Thus, in addition to the outdoor urban vs. nature conditions typically examined in past research, we also include an indoor control condition where participants do not view urban or nature elements. Past research has often compared an outdoor nature to an outdoor urban environment or has utilized an indoor environment for viewing nature vs. other stimuli but has not compared a true indoor control to both outdoor urban and outdoor nature environments.

Additionally, as activities involving low or moderate effort, such as walking, have been shown to be more effective for attention and affect than those involving high effort, such as jogging (Han, 2017), and as walking was shown to result in greater mental relaxation than sitting (Bailey and Kang, 2022), participants first complete a pre-test measure of affect before taking a walk in each of the three environments and then complete post-test measures of affect and cognitive performance. To further examine the varied effects of environment on cognition, we also utilize several cognitive tasks: moderate and high attentional tasks involving working memory and executive function, recall, and recognition memory. Finally, to address the possibility that null or inconsistent findings for cognitive tasks may be related to the nature environment not resulting in restoration or improvements in affect, we will also examine the restorative quality for each environment and changes in affect from pre-walk to post-walk. Consistent with past research and ART, we hypothesized (1) that the outdoor nature environment will be the most restorative and the indoor environment will be the least restorative and (2) consistent with ART and SRT, the outdoor nature environment will result in the greatest and the indoor environment will result in the smallest increase in positive affect and decrease in negative affect from pre-to post-walk. We also hypothesized (3) that those in the outdoor nature condition will perform better on moderate attention tasks than those in the outdoor urban and indoor conditions. Further, if simply being outside is beneficial, then the outdoor urban condition should also result in greater improvements in affect and better cognitive performance than the indoor control condition. However, if being outside is not beneficial, then the outdoor urban and indoor control conditions should not differ. Lastly, as findings for other cognitive tasks have been inconsistent, performance on the high attention and memory tasks will be analyzed for differences between conditions.

## Method

### Participants

Participants included 188 undergraduate students at a private Christian University with high access to natural environments. Students received course credit in an introductory psychology class for their participation in the study. See Table 1 for participant demographics including gender, race and ethnicity, age, and household income.

**Table 1:** Participant demographics (*N* = 188).

	*n*	%
Gender		
Cisgender male	68	36.2
Cisgender female	118	62.8
Nonbinary, Genderfluid, Nonconforming, or Other	2	1.0
Race and ethnicity		
White	126	66.7
Black or African American	9	4.8
Asian or Asian American	53	28.0
Latinx or Hispanic	29	15.3
American Indian or Alaskan Native	5	2.6
Native Hawaiian or Pacific Islander	2	1.1
	*M*	*SD*
Age	18.79	1.02
	*Median*	
Household income	$100,000 USD	

*N* = 63 (Outdoor Nature), 66 (Outdoor Urban), and 59 (Indoor). No participant demographics differed by condition, all *ps* > 0.24. Not all percentages total 100% as participants were not required to answer demographic questions, or (for Race and Ethnicity) may have selected more than one answer.

### Materials

#### Positive and negative affect scale (PANAS)

Participants rated how strongly they were currently experiencing 10 positive (e.g., inspired) and 10 negative (e.g., upset) feelings on a Likert-scale ranging from 1 (*very slightly or not at all*) to 5 (*extremely*). Scores could range from 10 to 50 for each positive (PA) and negative affect (NA) total. The PANAS (Watson et al., 1988) has demonstrated high reliability for both PA (0.88) and NA (0.87). Alpha in the current sample was 0.91 for the 10 PA items and was also 0.91 for the 10 NA items.

#### Restorativeness survey

The Perceived Restorativeness Scale (PRS; Pasini et al., 2014), consists of 11 items grouped into 4 factors of the restorative quality of natural environments: Being Away (e.g., “Places like this are a refuge from nuisances”), Fascination (“e.g., In places like this my attention is drawn to many interesting things”), Coherence (e.g., “There is a clear order in the physical arrangement of places like this”), and Scope (e.g., “That place is large enough to allow exploration in many directions”). Participants rate each item on a scale of 1 (*Strongly Agree*) to 7 (*Strongly Disagree*). The average response was calculated for the total scale. Alpha in the current sample was 0.93.

#### Walking environment

Participants completed a 20-min walk in one of three environments. The outdoor nature environment (see Appendix 1) was a wide dirt and gravel-packed path that followed a stream through trees, a canyon, and historical ruins in Solstice Canyon in Malibu, California; the natural features of this environment should allow for “soft fascination,” allowing attentional resources to recover. At the parking area near the trailhead, a shaded pavilion with picnic tables served as the location for completing all pre-and post-walk measures.

The outdoor urban environment was a busy road in Calabasas, California (see Appendix 1). There were many office and retail buildings on both sides of the 4-lane street and many cars driving along the street; the features of this environment should not allow attentional resources to recover. Participants were instructed to meet the researchers at the Calabasas Campus of Pepperdine University parking lot and completed all pre-and post-walk measures in a shaded courtyard area of the campus.

The indoor control location consisted of a research laboratory room on the Malibu campus of Pepperdine University. The room was divided with a treadmill (Nordic Track T 6.5S) on one side of the room. The other side, which was separated by a partial wall, contained a table and chairs for participants to complete all study measures. The room was intentionally kept free of any stimuli picturing natural or urban elements.

#### Cognitive measures

##### Memory

Participants completed a shortened variant of the Rey Auditory Verbal Learning Task (AVLT; Rey, 1964). The participants viewed a 15-item word list from Potter and Keeling (2005) at a rate of 1 s per word and were then prompted to type all the words they could recall for a measure of short-term recall memory. After approximately 15 min (during which time participants completed other measures; see procedure), participants were then presented with 30 randomly ordered words (15 of which were the original words and 15 of which were new) and were asked to indicate whether they had seen each word earlier. For recognition, the ability to discriminate between “old” and “new” was measured by d’.

##### Attention/working memory

Participants completed the Digit Span Forward (DSF) and Digit Span Backward (DSB) test (WAIS-IV; Wechsler, 2008). For DSF, conceptualized as a moderate attention task, participants viewed digit sequences at a rate of 1 s per digit and were required to type them in order after each sequence. Sequences were two to nine digits in length (two sequences of each length for a total of 16 sequences) and were presented in increasing length. For DSB, a high attentional load task, participants viewed digit sequences and were required to type them in backwards order (i.e., if the sequence was “3, 2” they were to report “2, 3”). There were 14 sequences, consisting of two sequences each of two to eight digits. The number of correct sequences was recorded, with a maximum score of 16 (DSF) or 14 (DSB).

##### Attention/executive function

Participants completed the Trail Making Test A (TMTA) and B (TMTB; Bowie and Harvey, 2006) on the Trail Making Test android app (Sacchi, 2020) installed on a provided research tablet. Both parts consisted of 25 circles distributed over the screen. In TMTA, a moderate attention task, the circles were numbered 1–25, and participants drew lines with their finger to connect the numbers in ascending order as quickly as possible, without lifting their finger from the screen. In TMTB, requiring higher attention, the circles included both numbers (1–13) and letters (A – L) and participants were instructed to alternate between the numbers and letters (i.e., 1-A-2-B-3-C, etc.). If a participant made an error, they heard a beep and then corrected the error. The total time to complete each test was measured. Before beginning Parts A and B, a practice version of each with 8 circles was presented; these practice versions were not scored.

### Procedure

**Figure 1:**
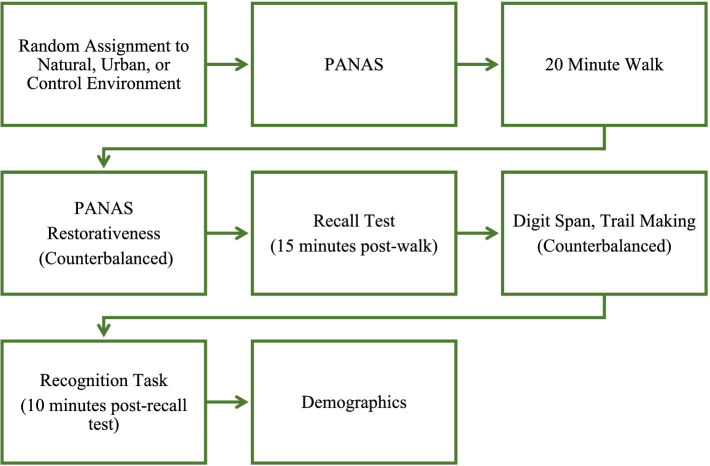
Experimental procedure.

This research was approved by the Institutional Review Board of Pepperdine University. The data were collected over the course of a Fall semester, from early September to early December. Prior to their participation, participants were randomly assigned to one of the three walking locations and were provided directions for where to meet the researcher. After arriving at the research location, participants gave informed consent and completed the initial affect measure (PANAS) via a Qualtrics survey on a provided Android tablet. Researchers then explained that participants would walk for 20 min and should adhere to a moderate effort (a 12–13 on the Borg Scale of Perceived Exertion; Borg, 1982) which was described as between a light and somewhat hard effort that should not elevate their breathing. For both the natural and urban environment, participants were given a simple timer that was pre-set to beep at 10 min and were instructed to turn around and return to the starting location when the 10 min had passed. For those in the indoor condition, no timer was needed as the treadmill displayed the elapsed time; participants were instructed to stop after 20 min. After the walk participants completed several survey measures in counterbalanced order, including a second PANAS, the Restorativeness Survey, and additional measures not reported here. These measures took approximately 15 min to complete. Next, participants completed the recall portion of the memory test, followed by the Digit Span and Trail Making cognitive tasks in random order, which took about 10 min to complete. Participants then completed the recognition portion of the memory task. Finally, participants completed the demographic questions and were thanked for their participation. See Figure 1 for procedure. This study was part of a larger data collection; the measures and hypotheses relevant to this study were pre-registered at https://osf.io/675ju?view_only=7df6b3e93af94afbb7c4fb2fdc1de705.

## Results

Preliminary analyses were conducted to determine whether systematic differences between the walking location groups on relevant demographics (see Table 1) existed. No gender differences between the groups were found, *X^2^* (4, *N* = 188) = 1.70, *p* = 0.79. Similarly, ANOVAs showed no differences in age or household income, all *ps* > 0.24. Participants who failed an attention check question or did not complete all measures (*N* = 5) were removed from further analyses.

### Restoration

To determine if the environments were different in restoration, we conducted a one-way ANOVA with the factor of walking environment. As predicted, there was a significant effect of location (see Table 2 for means and standard deviations of outcome variables and Table 3 for summary statistics); *post-hoc* (Bonferroni) tests revealed that each location differed significantly from the other two locations, all *ps* < 0.001, with the outdoor nature environment as the most restorative, followed by the outdoor urban environment; the indoor environment was the least restorative.

**Table 2:** Means and standard deviations for outcome measures.

Positive affect	Pre-walk		Post-walk	
	*M*	*SD*	*M*	*SD*
Outdoor nature	28.12	7.48	34.52	8.03
Outdoor urban	27.05	7.56	30.00	9.36
Indoor	28.47	7.85	31.14	7.86

Negative affect	Pre-walk		Post-walk	
	*M*	*SD*	*M*	*SD*
Outdoor nature	15.63	5.09	12.57	3.13
Outdoor urban	14.75	4.14	13.31	3.98
Indoor	15.00	5.00	13.78	4.79

Perceived restoration	*M*	*SD*
Outdoor nature	61.61	8.26
Outdoor urban	50.55	11.00
Indoor	34.36	12.24

Memory	Recall		Recognition d’	
	*M*	*SD*	*M*	*SD*
Outdoor nature	6.78	1.66	2.10	1.08
Outdoor urban	6.14	2.34	1.74	0.91
Indoor	6.53	2.19	1.93	1.23

Attention/working memory	Digit span forwards (DSF)		Digit span backwards (DSB)	
	*M*	*SD*	*M*	*SD*
Outdoor nature	9.37	1.82	8.63	1.92
Outdoor urban	10.12	2.21	8.98	2.28
Indoor	9.44	2.52	8.00	2.74

Attention/executive function	Trail Making A (TMTA)		Trail Making B (TMTB)	
	*M*	*SD*	*M*	*SD*
Outdoor nature	50.92	5.84	92.81	58.30
Outdoor urban	60.56	5.60	103.59	73.16
Indoor	52.42	5.84	101.68	58.42

Cognitive tasks and perceived restoration were assessed only once, post-walk. Trail Making Part A and B are represented as time in seconds.

**Table 3:** ANOVA summary statistics for analyses.

	*F*	*df*	*p*	*η_p_^2^*
Perceived restoration**	87.41	2, 180	<0.001	0.52
Positive affect				
Time**	71.05	1, 180	<0.001	0.28
Location	2.21	2, 180	0.11	0.02
Time X location**	6.32	2, 180	0.002	0.07
Outdoor nature**	80.99	1, 59	<0.001	0.58
Outdoor urban**	13.16	1, 63	0.001	0.17
Indoor**	8.18	1, 58	0.006	0.12
Negative affect				
Time**	46.91	1, 180	<0.001	0.21
Location	0.14	2, 180	0.87	0.002
Time X location*	4.32	2, 180	0.02	0.05
Outdoor nature**	33.23	1, 59	<0.001	0.36
Outdoor urban**	9.26	1, 63	0.003	0.13
Indoor**	7.82	1, 58	0.007	0.12
Memory				
Recall	1.49	2, 180	0.23	0.02
Recognition (d’)	1.72	2, 180	0.18	0.02
Attention				
Digit span forwards (DSF)	2.26	2, 180	0.11	0.02
Digit span backwards (DSB)	2.76	2, 180	0.07	0.03
Executive function				
Trail making A (TMTA)	0.83	2, 179	0.44	0.01
Trail making B (TMTB)	0.49	2, 179	0.49	0.01

*p < 0.05; **p < 0.01.

### Affect

To test the hypothesis that walking in nature compared to other environments would result in a greater increase in positive affect and decrease in negative affect from pre-to post-walk, we conducted a 2 × 3 repeated measures mixed ANOVA with the within-subjects factors of Time (pre-walk, post-walk) and the between subject factor of walk environment (Location: outdoor nature, outdoor urban, indoor) for both PA and NA. See Table 2 for means and standard deviations for PA and NA as a function of time and location and Table 3 for summary statistics.

#### Positive affect

For PA, there was a significant effect of time, such that positive affect was higher post-walk than pre-walk. There was no significant effect of location. As predicted, there was a significant time X location interaction. Analyses of simple main effects showed that while all three conditions increased in PA from pre-to post-walk, the effect of the walk was large in the outdoor nature group, moderate in the outdoor urban group, and small in the indoor group: while all groups were equivalent on PA pre-walk, the outdoor nature group had the highest increase and the indoor group the lowest increase in PA from pre-to post walk.

#### Negative affect

For NA, there was a significant effect of time, such that NA decreased from pre-to post-walk. There was no main effect of location. As predicted, there was a significant time X location interaction. Analyses of simple main effects showed that while all three conditions decreased in NA from pre-to post-walk, the effect of the walk was large in the outdoor nature group and small in both the outdoor urban group and indoor group: while all groups were equivalent on NA pre-walk, the outdoor nature group decreased the most and the indoor group the least in NA from pre-to post-walk.

### Cognitive measures

To test the hypothesis that walking in nature would result in the best performance on the moderate attention tasks (DSF, TMTA) and to test for differences on the high attention (DSB, TMTB) and memory (recall, recognition) tasks, we conducted a one-way ANOVA with the factor of walk environment (outdoor nature, outdoor urban, indoor) for each measure; see Tables 2 3 for all *M*, *SD*, and summary statistics. No differences were found for any of these cognitive measures.

## Discussion

As hypothesized and consistent with prior literature (Bowler et al., 2010; McMahan and Estes, 2015; Norwood et al., 2019; Zhang et al., 2020), those in the outdoor nature condition increased in positive affect and decreased in negative affect more than the outdoor urban and indoor locations. Also as hypothesized, the outdoor nature location was rated the most restorative and the indoor location was the least restorative. Importantly, the inclusion of both an outdoor urban and an indoor control group revealed that differences in affect between the outdoor nature and indoor conditions may be partially, but not entirely, due to the effect of being outside: changes in affect were strongest for the outdoor nature and weakest for the indoor, with the outdoor urban condition in between. Past research has often compared an outdoor nature to an outdoor urban environment or compared viewing urban and nature stimuli while indoors. Less frequent is a comparison of an outdoor nature to an indoor environment; however, past research has not included both an outdoor urban and indoor control (where participants do not view urban or nature stimuli) in the same study. These findings imply that while nature exposure does have effects on affect above and beyond the effects of simply being outside, urban outdoor environments result in larger effects on positive affect than being inside. Future research should continue to tease apart effects of natural environments from effects of being outdoors.

Past research has shown generally consistent findings of attentional benefits from exposure to natural environments (Ohly et al., 2016; Stevenson et al., 2018) and inconsistent or null effects of natural environments on other measures of cognition (Stevenson et al., 2018; Trammell and Aguilar, 2021). This research, however, demonstrated no effects of environment on cognition. The null findings for tasks high in attentional demand are consistent with Trammell and Aguilar (2021), but the lack of differences for even moderate measures of attention is surprising and suggests that differing attentional demands does not account for the inconsistent effects of nature on cognition in previous literature. Further, these results do not support that being outside is beneficial for cognition, as all three groups (outdoor nature, outdoor urban, indoor control) performed similarly. These findings only partially support ART (Kaplan, 1995; Berman et al., 2008), which proposes that natural environments would lead to both restoration and improvement in attentional tasks. Similarly, these results also do not fully support SRT (Ulrich et al., 1991), as while a reduction in negative affect was found, differences between conditions in cognitive performance were not significant. This implies that in research where cognitive effects have been found, factors other than or in addition to restoration and affect may be involved; precisely what these factors may be has yet to be determined. Participants in this study were not directed to focus attention or engage in their environment in any way. As prior research has shown active engagement with the environment improves the effect of natural environments on attention (Lin et al., 2014; Pasanen et al., 2018), it is possible that including such instructions may have resulted in differences between environmental conditions. However, active engagement does not appear to be necessary, and in some cases may counteract, the restorative effects of natural environments (Macaulay et al., 2022).

It is also possible that no cognitive effects were observed as participants were attending a college that was located in a natural environment surrounded by mountains and the Pacific Ocean. It would be difficult to not be exposed to natural environments on a daily basis, which may dilute their impact. However, affective and restorational benefits were still observed, suggesting that frequent exposure does not negate all benefits of an acute exposure to a natural environment. Although past research has demonstrated that those who live near natural environments (Cox et al., 2017; White et al., 2021) and adolescents with greater exposure to natural environments (Li et al., 2018) score better on a number of measures of mental health than those who do not, future research could compare the cognitive effects of an acute nature exposure in those with high vs. low regular exposure to nature. A related possibility is that the environments chosen for this study were sufficient to induce differences in affect, but not in cognition. Prior research has shown that extraordinary (grand and wild) nature leads to greater effects on awe, mood, and other emotions than mundane nature (Joye and Bolderdijk, 2015). Although cognition was not measured by Joye and Bolderdijk (2015), it is reasonable to infer that cognitive effects of nature may likewise be greater in extraordinary than mundane nature. The environment chosen for the outdoor nature condition in this study did not contain the towering mountains or rushing rivers that are typical of extraordinary nature environments. For those with high access to natural environments, it may be that extraordinary environments are necessary to elicit differences in cognition.

A limitation of our sample is that it was not representative of the general U.S. population. Notably, our participants had higher median household income ($100,000) than the general population ($74,580; U.S. Census Bureau, 2023). Such higher income combined with the privilege of frequent exposure to natural environments indicates that the results from these participants may not generalize to a larger, more diverse population. Another limitation is that the natural environment was located close to the college campus, and thus was similar to the natural areas surrounding the campus. If frequent exposure does in fact reduce the cognitive effects of natural environments, then choosing a natural environment that was more different from the environments to which our participants were frequently exposed may have resulted in larger effects. Additionally, cognitive measures were performed only once, post-task. While our results clearly show no differences in post-walk cognition between conditions in our randomly assigned groups, we are not able to ascertain if there were differences in *changes in* cognition from pre to post-walk between locations.

In conclusion, these results demonstrate that exposure to nature improved affect, but did not affect cognition. It is possible that everyday exposure to nature may prevent the cognitive effects of restorative environments, but does not negate the affective benefits, suggesting that the affective benefits may be more robust than cognitive effects. More research is needed to determine the factors that may result in cognitive improvements in some natural environments but not others. We recommend that those interested in low-cost and low-effort interventions should consider that spending time outdoors, particularly in nature, is an effective and reliable way to improve affect but may not necessarily result in cognitive benefits.

## Data availability statement

The raw data supporting the conclusions of this article will be made available by the authors, without undue reservation.

## Ethics statement

The studies involving humans were approved by Pepperdine University, Seaver IRB. The studies were conducted in accordance with the local legislation and institutional requirements. The participants provided their written informed consent to participate in this study.

## Author contributions

JT: Writing – original draft, Writing – review & editing. JH: Writing – review & editing. EK-M: Writing – review & editing.

## Appendix 1

**Table:**
